# Alpha and Beta Naphthol

**Published:** 1901-03-15

**Authors:** 


Alpha and Beta Naphthol.—According to *Maximowitch*, the preference for medicinal use of Beta-Naphthol is not well founded. He states that alpha-naphthol is three times as antiseptic as beta-naphthol, and one-third as poisonous. Beta-naphthol is also more irritating to the mucous membrane of the mouth and stomach. He therefore always employs alpha-naphthol. —*Therapeutic Gazette.* [This may be accounted for by the fact that alpha-naphthol is less soluble than beta. This would make it a better antiseptic, but a poorer germicide.—Ed.]

